# Comparison of Eleven Methods for Genomic DNA Extraction Suitable for Large-Scale Whole-Genome Genotyping and Long-Term DNA Banking Using Blood Samples

**DOI:** 10.1371/journal.pone.0115960

**Published:** 2015-01-30

**Authors:** Androniki Psifidi, Chrysostomos I. Dovas, Georgios Bramis, Thomai Lazou, Claire L. Russel, Georgios Arsenos, Georgios Banos

**Affiliations:** 1 Animal Production Laboratory, School of Veterinary Medicine, Aristotle University of Thessaloniki, Thessaloniki, Greece; 2 The Roslin Institute and Royal (Dick) School of Veterinary Studies, University of Edinburgh, Edinburgh, United Kingdom; 3 Microbiology and Infectious Diseases Laboratory, School of Veterinary Medicine, Aristotle University of Thessaloniki, Thessaloniki, Greece; 4 Food safety Laboratory, School of Veterinary Medicine, Aristotle University of Thessaloniki, Thessaloniki, Greece; 5 Department of Clinical Veterinary Sciences, University of Bristol, Langford House, Langford, Bristol, United Kingdom; 6 Scotland’s Rural College, Edinburgh, United Kingdom

## Abstract

Over the recent years, next generation sequencing and microarray technologies have revolutionized scientific research with their applications to high-throughput analysis of biological systems. Isolation of high quantities of pure, intact, double stranded, highly concentrated, not contaminated genomic DNA is prerequisite for successful and reliable large scale genotyping analysis. High quantities of pure DNA are also required for the creation of DNA-banks. In the present study, eleven different DNA extraction procedures, including phenol-chloroform, silica and magnetic beads based extractions, were examined to ascertain their relative effectiveness for extracting DNA from ovine blood samples. The quality and quantity of the differentially extracted DNA was subsequently assessed by spectrophotometric measurements, Qubit measurements, real-time PCR amplifications and gel electrophoresis. Processing time, intensity of labor and cost for each method were also evaluated. Results revealed significant differences among the eleven procedures and only four of the methods yielded satisfactory outputs. These four methods, comprising three modified silica based commercial kits (Modified Blood, Modified Tissue, Modified Dx kits) and an in-house developed magnetic beads based protocol, were most appropriate for extracting high quality and quantity DNA suitable for large-scale microarray genotyping and also for long-term DNA storage as demonstrated by their successful application to 600 individuals.

## Introduction

The successful completion of the Human Genome Project and the achievement of similar goals in other species have generated a huge amount of freely available information about the genomic sequence of different organisms, opening the door to a post-genomic era where new challenges arise [1,2]. This new era is also characterized by the development of new technologies which enable the study of thousands of genes and/or molecular markers at once. Such a technology is based on DNA microarrays, which is a multiplex technique used for rapid, large-scale genotyping. This technique has fast become a standard approach in molecular biology research and clinical diagnostics [3]. Microarrays have already been successfully applied in as diverse scientific studies as cell biology, molecular microbiology, cancer genetics, genetic and metabolic disorders, infectious diseases, drug discovery, host-pathogen interaction, population genetics, linkage analysis, genetic improvement of livestock species, evolutionary biology, detection of food-borne pathogens, stress responses, forensic analysis and toxicological research [3–9].

In the last few years, further enormous advances in genotyping technology have been taking place with the development of the next generation sequencing (NGS) technologies. Whole genome sequencing provides information on a genome that is orders of magnitude larger than that provided by DNA microarrays [10]. To date, these technologies have been applied in a variety of contexts, including whole genome sequencing, de novo genome sequencing, exome sequencing, targeted resequencing, cancer cell sequencing, de novo transcriptome sequencing, RNA sequencing, small RNA sequencing, metagenomic sequencing and microbial strain screening, among others [11–18] (http://www.beckmangenomics.com/genomic_services/next_generation_sequencing/). Although NGS platforms are improving at a very quick rate, thereby reducing costs by a factor of two to three each year, the cost is still too high for routine large-scale sequencing of whole genomes for scientific research [19]. At this point, next generation platforms are usually used as complementary to microarray analysis.

Microarray technology has been improved significantly in that period, in terms of diminished cost and sample requirement, and has yielded increased data density and quality [20]. However, it still remains a complex process that is prone to technical difficulties if reagents and input material are not of suitable quality [21,22]. The first crucial step for microarray analysis is considered to be DNA extraction and quality control of the extracted nucleic acids. Whole-genome microarray analysis continues to require an input DNA mass that is at least 100 times larger than that required for simple PCR testing and requires very pure DNA that is double stranded with a length span at least 5 times longer than required for most PCR reactions [23]. Usually, a DNA quantity of 2.5 to 3.0 μg is necessary depending on the array size and platform used (http://www.ark-genomics.org/news/edinburgh-genomics). However, when other panels and techniques are used for whole genome genotyping, like KASP genotyping, a higher quantity of DNA, up to 6.0 μg, is required (http://www.lgcgenomics.com/genotyping/kasp-genotyping-chemistry/genotyping-panels). Similarly, in the case of NGS, DNA quantity requirements differ depending on the genotyping aim and the platform used. For whole genome *de novo* sequencing, which is used to sequence uncharacterized genomes where there is no reference sequence available or known genomes where significant structural variation is expected like in cancer cells, a very high DNA quantity is required, usually from 30 to 60 μg depending on the platform. For whole genome sequencing, usually a quantity above 10 μg, ideally 20 μg DNA is desirable, while for targeted resequencing of custom regions of interest a lower DNA quantity of about 3 to 6 μg is used [12] (http://genepool.bio.ed.ac.uk/illumina/samples.html). A minimum concentration of 50 ng/μl is also necessary in both microarray and NGS analysis. Picogreen assay with Qubit platform is considered to be the method of choice for DNA quantification. Implementation of quantification methods other than Picogreen may lead genotyping companies to ask for more concentrated and higher amounts of DNA for the analysis (http://genepool.bio.ed.ac.uk/illumina/samples.html). A gel photo documenting high quality DNA is required to accompany the samples, as well. Although the different NGS and microarray platforms have specific requirements regarding DNA quantity, purity and integrity in order to achieve reliable genotyping data, no specific guidance on the protocol of choice is given by the genotyping centers. The need for robust methods that produce a representative, non-biased source of nucleic acid material from the genome under investigation is acknowledged [12].

Another important aspect regarding DNA extraction protocols and advanced genotyping analysis is the suitability of the extracts for long term DNA-banking. Usually DNA extracts have to be stored until all samples are collected, which differs among the studies, and until the genotyping centers have capacity available. Moreover, there is an increased interest in the creation of DNA banks since sample collection and DNA extraction are laborious, expensive and time consuming procedures. Storage tests carried out by the DNA Bank Network revealed that high purity of extracted DNA must be ensured, since secondary compounds and heavy metal ions can result in highly reactive intermediates causing all sorts of DNA damage [24,25].

Although, the selection of an appropriate DNA extraction method plays a pivotal role in the success of genome-wide studies and long term DNA-banking, there are no established standard operating procedures for genomic DNA extraction. Moreover, there are no published reports on simultaneous comparisons of the efficiency of different genomic DNA extraction procedures for microarray analysis or NGS applications, and only a few studies in the literature that compare different extraction protocols for microbial DNA suitable for microarrays analysis [21,26–29].

The objective of this work was to evaluate eleven different methods for extraction of genomic DNA from ovine blood samples in terms of DNA quantity, concentration, purity, integrity and real-time PCR suitability, as well as utility and applicability for subsequent DNA microarray genotyping and long–term storage.

## Materials and Methods

### Resource Population and Sample Collection

At first, 11 blood samples were taken from each of 16 ewes of the Chios dairy breed raised in an experimental flock. These samples were used to evaluate the DNA extraction methods described next. Peripheral blood samples were collected in 9 ml K_2_EDTA Vacutainer blood collection tubes (BD diagnostics) by jugular venepuncture. These samples were inverted to mix and prevent clotting and immediately placed in isothermic boxes and transferred to the laboratory. Individual blood samples from the same animal where mixed together and then divided again in order for each blood sample to contain the same amount of leucocytes. At the end of the procedure, all 16 animals had DNA extracted with each one of 11 DNA extraction methods described in detail in the next section.

Three of the DNA extraction methods (Nucleospin Blood, Nucleospin Blood L, Nucleospin Blood XL, Macherey-Nagel, Duren, Germany) used whole blood as source of genomic DNA while the rest of them used buffy coat (Table 1). In the latter cases, buffy coat was prepared by spinning whole blood at 3,000 g for 10 min in an Eppendorf (5415R) centrifuge (Hamburg, Germany) at room temperature to separate the blood into its plasma, leukocyte and erythrocyte fractions. The buffy coat was removed and dispersed in 700 μl of red cell lysis buffer (25 mM NaHCO_3_, 0.3 M NH_4_Cl, 5 mM EDTA). A second centrifugation at 3,000 g for 10 min at room temperature followed. The liquid was discarded and the leucocyte pellet was dispersed in different buffers depending on the DNA extraction method followed.

**Table 1:** Eleven DNA extraction methods and descriptive statistics of the measurements used to evaluate the respective quality of DNA extracted.

**Method** *	**OD 260/280**			**OD 260/230**			***C. coli* Ct values**		
	**Median**	**Range**	**SD**	**Median**	**Range**	**SD**	**Median**	**Range**	**SD**
**Nucleospin Blood (200 μl WB, SC)**	1.76	1.6–1.8	0.68	1.83	1.0–2.1	0.30	29.65	28.8–30.4	0.55
**Nucleospin Blood L(2 ml WB, SC)**	1.69	1.2–1.8	0.21	1.30	1.1–1.9	0.53	29.64	28.7–30.5	0.63
**Nucleospin Blood XL (10 ml WB, SC)**	1.70	1.3–1.8	0.12	1.35	0. 9–1.8	0.32	29.68	28.6–30.6	0.62
**Nucleospin Blood-Buffy Coat (9 ml BC, SC)**	1.94	1.8–2.0	0.06	2.14	1.9–2.2	0.11	29.68	28.7–30.2	0.57
**Nucleospin Tissue-Buffy Coat (9 ml BC, SC)**	1.80	1.7–1.9	0.07	2.13	1.6–2.5	0.30	29.50	28.7–30.2	0.60
**Modified Blood (9 ml BC, SC)**	1.87	1.7–1.9	0.05	2.30	1.9–2.2	0.19	29.57	28.7–30.2	0.56
**Modified Tissue (9 ml BC, SC)**	1.86	1.7–2.0	0.07	2.13	1.6–2.4	0.30	28.83	29.5–30.3	0.56
**Modified Dx (9 ml BC, SC)**	1.82	1.6–1.9	0.08	2.11	1.8–2.3	0.15	30.20	28.4–30.5	0.72
**Phenol – Chloroform (9ml BC, FC)**	1.67	1.5–1.8	0.11	2.04	1.3–2.3	0.28	30.20	26.7–32.7	1.39
**Charge-Switch (4.5 ml BC, B)**	1.47	1.3–1.7	0.12	0.75	0.6–1.2	0.14	30.12	28.7–30.4	0.62
**In-house (4.5 ml BC, B)**	1.72	1.6–1.9	0.09	1.88	1.7–2.1	0.13	30.07	28.8–30.3	0.55

* Volume, DNA source and technology used are in parentheses; WB = whole blood, BC = buffy coat, SC = silica column, B = beads, FC = Phenol-Chloroform extraction

In addition, 600 blood samples were collected from as many Chios ewes (different from the 16 animals mentioned previously) raised in five different flocks in the northern part of Greece. These samples were used for a large-scale application and further evaluation of the DNA extraction methods of choice.

During sampling animals were handled by qualified veterinarians. Permission to qualified veterinarians to perform blood sampling was established by the National (Greek) Legislature for the Veterinary Profession, No. 344/29-12-2000. The study was approved by the Ethics and Research Committee of the Faculty of Veterinary Medicine, Aristotle University of Thessaloniki, Greece, which monitors and approves all experimental protocol carried out in the flock in the experimental farm. This farm is a model commercial farm equipped with modern facilities. Housing is designed to provide optimal welfare conditions for raising sheep in terms of space availability and ventilation in the sheep shed. Permit for access and use of the experimental flock was obtained from the Ethics and Research Committee of the Faculty of Veterinary Medicine, Aristotle University of Thessaloniki, Greece. Permits for the commercial farms were granted by the farm owners, who were members of the Chios Sheep Breeders’ Cooperative “Macedonia”.

The study did not involve endangered or protected species. The location of the farms was in the Northern Greece (latitude 41oN, 23oE).

### DNA Extraction Methods

Eleven different DNA extraction methods were evaluated using whole blood and buffy coat obtained from the 16 ewes. All methods are listed in Table 1.

Three commercially available kits, Nucleospin Blood, Nucleospin Blood L, Nucleospin Blood XL (Macherey-Nagel, Duren, Germany) were tested. The amount of whole blood used as source of genomic DNA was 200 μl, 2 ml and 10 ml, respectively. DNA extraction was performed according to the manufacturer’s instructions.

Nucleospin Blood-Buffy Coat and Nucleospin Tissue-Buffy Coat methods (Table 1) were direct applications of extraction kits Nucleospin Blood and Nucleospin Tissue (Macherey-Nagel, Duren, Germany), respectively, with a buffy coat of 9 ml blood being used instead of 200 μl whole blood. The leucocytes were re-suspended in 200 μl PBS and extraction was performed according to the manufacturer’s instructions. At the end, the purified DNA was eluted from the nucleospin column in a 150 μl elution buffer (EB).

In Modified Blood, Modified Tissue and Modified DX methods (Table 1), Nucleospin Blood, Nucleospin Tissue and Nucleospin Blood Dx commercial kits (Macherey-Nagel, Duren, Germany), respectively, were modified in order to increase DNA recovery and purity. The key modifications introduced to the commercial kits consisted of sample pretreatment to eliminate PCR inhibitors and thus increase DNA yield. Specifically, buffy coat was treated with increased volumes of lysis buffers and proteinase K. In addition, duration of incubation with proteinase K was increased to eliminate the amounts of cell debris, proteins and lipids. A chloroform step to remove lipids and other insoluble solids was added as well. More specifically, buffy coat of 9 ml blood was used as a source of DNA. Leucocyte cells were re-suspended in 200 μl PBS. Increased volumes of proteinase K (37.5 μl instead of 25 μl) and lysis buffers (270 μl instead of 180 μl) from each kit were added, and the mixture was incubated first in 56°C for 1.5 hours and then in 70°C for 15 min. Subsequently, 500 μl of chloroform were added and the mixture was vigorously rotated for 20 min. After centrifugation at 16,000 g for 10 min at 4°C using an Eppendorf (5415R) centrifuge, the aqueous phase was transferred to a tube containing 320 μl of absolute ethanol. The mixture was then applied to nucleospin columns (different for each kit) and DNA was absorbed onto the nucleospin silica gel membrane during one centrifugation at 11,000 g for 1 min. The silica was washed once using 500 μl of a guanidine containing buffer (BW) and then twice using 400 μl of an ethanol containing buffer (B5). The purified DNA was eluted from the nucleospin column in a 200 μl elution buffer (EB).

Phenol-Chloroform method (Table 1) was a standard phenol-chloroform based extraction protocol containing proteinase K [30]. Buffy coat of 9 ml blood was used. The only modifications to that protocol were the addition of two extra steps in the end of the protocol. Briefly, after the suspension of DNA in the elution buffer, 2 μl RNAse A (5 mg/ml Invitrogen, Carlsband, CA) were added to digest the remaining RNA and the elution was extracted first with 200 μl chloroform and afterwards with 200 μl PEG/NaCl (PEG 20%, NaCl 2.5M). The aqueous phase was transferred and the DNA was precipitated at room temperature after the addition of 0.5 ml of 75% ethanol. By a final centrifugation the DNA pellet was dried and re-suspended in 50 ml DPCW and incubated overnight in 4°C.

Another commercially available kit, the ChargeSwitch gDNA Tissue Mini (Invitrogen, Carlsbad, CA), was also tested (Table 1). Buffy coat of 4.5 ml blood was used as a source of genomic DNA. The DNA extraction was performed according to the manufacturer’s instructions.

The last method (Table 1) was an In-house developed protocol based on the lysing and nuclease-inactivating properties of two chaotropic agents, guanidinium hydrochloride (GuHCl) and guanidinium thiocyanate (GuaSCN), together with the use of magnetic beads as the affinity matrix. Different blood quantities were tested for buffy coat preparation and the quantity of 4.5 ml was chosen because it resulted in better DNA quantity and quality. In brief, leucocyte cell pellet, coming from buffy coat, was resuspended in 250 μl PBS. Volumes of 250 μl lysis buffer A (50mM Tris- HCl, 50mM EDTA, 4M GuHCl, 10 mM CaCl_2_, 1% v/v Triton X-100, 2% N-Lauroyl-Sarcosine, pH = 7.5) and 50 μl of proteinase K (22.4 mg/ml, MERK) were added and the lysate was homogenized and incubated in 56°C for 1.5 h. A volume of 600 μl of lysis buffer B (50 mM Tris-HCl, 25 mM EDTA, 6 M GuaSCN, 3% v/v Triton X-100, 6% N-Lauroyl-Sarcosine, pH = 5.5) was added and the suspension was incubated at 70°C for 10 min. 500 μl of absolute isopropanol was added to the lysate. Then, 40 μl (50 mg/ml) of magnetic silica particles, PMSi-H1.0–5 (Kisker Biotech GmbH, Steinfurt, Germany) were added for DNA binding. The samples were homogenized and placed to MagnaRack (Invitrogen, Carlsbad, CA). Subsequently, the magnetic beads were washed three times starting with 700 μl wash buffer A (25 mM Tris-HCl, 4 M GuHCl, 30% v/v absolute isopropanol, pH 6.6), then twice with 500 μl wash buffer B (10 mM Tris-HCl, 100 mM NaCl, 80% v/v absolute isopropanol, pH 6.6). As a final step, the magnetic beads were transferred to new 2 ml tubes and incubated at room temperature for 10 min with open caps. DNA was recovered in 1200 μl TE buffer sequentially, each time using 600 μl TE buffer (10 mM Tris-HCl, pH = 9) preheated in 65°C.

### Evaluation of DNA Extraction

#### Spectrophotometer and Qubit Measurements

Purity of DNA extracted with each method was assessed using an Eppendorf Biophotometer. The ratio of absorbance at 260 nm and 280 nm was used to assess protein contamination while the ratio of absorbance at 260 nm and 230 nm was calculated to assess guanidine contamination. Both spectrophotometric measurements constituted criteria for DNA quality assessment with higher values associated with better DNA purity.

Quantity of DNA extracted by the different methods was assessed using Qubit 2.0 fluorometer (Invitrogen, Life technologies). The Qubit fluorometer calculates concentration based on the fluorescence of a dye which binds to double stranded DNA (dsDNA). The Qubit fluorometer picks up this fluorescence signal and converts it into a DNA concentration measurement using DNA standards of known concentration. Qubit dsDNA BR Assay Kit was used for the DNA quantification.

Based on DNA concentration derived from the Qubit measurements and the volume of the DNA extract, total DNA yield was calculated with a simple multiplication.

#### Gel Electrophoresis

The integrity of DNA extracted by each method was assessed by gel electrophoresis [31,32]. Specifically, 1 μl of each DNA extract was analysed in a 1.5% agarose gel containing 0.5% ethidium bromide and was visualized by U.V. illumination.

#### Real-time PCR

A real-time PCR targeting the ovine prion protein gene (PRNP) was used to assess presence of amplifiable DNA in extracts of blood samples. The set of primers (amplifying a 168 bp PRNP genomic region), amplification reaction set up and thermo-cycling conditions described in a previous study [30] were applied here, too. Ct values were used to assess the amount of amplifiable DNA obtained. Smaller Ct values are desirable in this regard.

A second real-time PCR protocol was applied to assess the ability of the different genomic DNA extraction protocols to remove PCR inhibitors from blood samples [26,28]. The presence of PCR inhibitors in the genomic DNA extracts was tested by spiking 1000 bacterial genomic copies DNA (*Campylobacter coli C. coli*, strain ATCC 43478) into 100 ng and 1000 ng of sheep DNA extracts, respectively, followed by real-time PCR amplification of hydroxymethyltransferase gene (glyA). Real-time PCR amplifications [33] were performed using a Biorad CFX96 Real-time System.

All samples were run in triplicates while in every PCR trial, three controls containing only the *C. coli* DNA spike (without sheep DNA), were included. Ct values obtained in the process were used to assess the presence of PCR inhibitors. Specifically, the resultant inhibition of the amplification was assessed in comparison to the non-spiked control.

### Statistical Analysis

The 11 different DNA extraction methods were assessed with the model: Y_ij_ = μ + p_i_ + s_j_ + e_ij_, where Y_ij_ = DNA score by evaluation criterion for the i^th^ extraction method and j^th^ animal, μ = overall mean, p_i_ = effect of i^th^ DNA extraction method (i = 1–11), s_j_ = effect of the j^th^ animal (j = 1–16) and e_ij_ = random residual.

Each DNA evaluation criterion [two spectrophotometric measurements (O.D. 260/280, O.D. 260/230), DNA concentration, total DNA yield, and two real-time PCR results (Ct PRNP, Ct glyA)] was assessed in a separate analysis.

A Bonferroni adjustment for multiple testing was implemented in the comparison of different DNA extraction methods for each evaluation criterion. Statistical significance level was set at 0.05. All analyses were performed using the statistical package ASREML [34].

### Large-Scale Application, Long-Term DNA Storage and Microarray Genotyping

Based on the results of the statistical analysis, the modified methods (Modified Blood, Modified Tissue, Modified Dx kits) and the In-house developed protocol were selected to be further tested in order to assess their robustness and large-scale applicability for long-term storage and microarray analysis. Specifically, genomic DNA was extracted from 600 Chios ewes, using Modified Blood for 150 of them, Modified Tissue for another 150, Modified Dx for 200 and the In-house protocol for the remaining 100 ewes. The quantity and the purity of DNA extracts were assessed using a spectrophotometer (Eppedorf Biophotometer) and the samples were stored for 3 years at −20°C. At the end of this period, quality (O.D. 260/280 and O.D. 260/230) of the DNA was measured using a Nanodrop ND-1000 spectral photometer (Nanodrop Technologies, Wilmington, DE, USA) and quantity was measured using Qubit 2.0 fluorometer and Quant-iT PicoGreen dsDNA Assay Kit (Invitrogen, Life technologies). Integrity of DNA was tested by gel electrophoresis (1% agarose gel). The same model as above was used to assess differences in the large-scale performance of the four extraction protocols. Since there was only one observation per animal, the ewe effect was not included in the model in these analyses.

Subsequently, DNA extracts from all samples were diluted appropriately so the same quantity (2.5 μg) of DNA per sample would be available for microarray genotyping. Genotyping, which took place three years after the extraction of the DNA, was based on a customized 960 SNP whole-genome array (Illumina) including polymorphisms that had been previously found to have a significant effect on mastitis traits in three other breeds in three different countries [35,36].

The four extraction methods were then evaluated further based on the genotyping quality at each SNP locus assessed by the corresponding call rate. Call rate is defined as the proportion of genotype calls for a SNP assigned a genotype other than unknown [37]. Values range from 0 to 100% and higher values are desirable. Two genotyping quality measurements were used as criteria for the evaluation of the DNA extraction methods: average call rates per animal across all SNP and individual SNP call rates. Each genotyping quality criterion was assessed separately with a model including the effects of overall mean, DNA extraction method (1–4) and random residual. In the analysis of individual SNP call rates, the random effect of ewe was also included.

As before, a Bonferroni adjustment for multiple testing was implemented. Statistical significance level was set at 0.05. All analyses were performed using the statistical package ASREML.

## Results and Discussion

### Method Comparison

Descriptive statistics of all 11 methods for the two spectrophotometric measurements, the glyA real-time PCR results, the DNA concentration, the total DNA yield and the PRNP real-time PCR results based on application to 16 ewes are summarized in Tables 1 and 2, respectively. Marginal means for each extraction protocol and comparisons between protocols derived from the statistical analyses are shown in Table 3.

**Table 2:** Descriptive statistics of the measurements used to evaluate quantity of DNA extracted by 11 different methods.

**Method**	**DNA concentration (ng/μl)**			**Total DNA yield1 (μg)**			**PRNP Ct values**		
	**Median**	**Range**	**SD**	**Median**	**Range**	**SD**	**Median**	**Range**	**SD**
**Nucleospin Blood**	55.6	17–102	19	5.56	1.7–10	2	24.7	23.0–27.4	1.2
**Nucleospin Blood L**	112	18–377	102	22.5	4–75	20	23.9	22.2–28.1	2.6
**Nucleospin Blood XL**	163	61–322	71	326	122–644	143	23.2	21.4–25.7	1.2
**Nucleospin Blood Buffy Coat**	64.1	24–119	32	12.8	3.72	6	23.2	21.8–26.3	1.4
**Nucleospin Tissue Buffy Coat**	49.2	20–131	37	7.38	3–19	5	24.5	22.9–27.5	1.3
**Modified Blood**	103	52–172	46	15.5	10–26	7	24.0	22.3–25.4	0.9
**Modified Tissue**	104	61–360	67	21.6	12–72	13	23.3	19.0–26.2	1.7
**Modified Dx**	231	61–768	185	46.2	12–153	37	23.1	21.9–24.7	0.8
**Phenol—Chloroform**	260	4–585	154	13	0.2–29	7	24.5	21.7–29.3	2.5
**Charge-Switch**	7.1	3–10	2	1.85	0.7–4	1	26.0	23.7–29.1	2.0
**In-house**	288	141–592	129	346	169–710	155	23.3	21.6–26.6	1.4

**Table 3:** Statistical comparison of 11 genomic DNA extraction methods applied to 16 animals.

**Method (total DNA volume (μl) in parenthesis**	**OD 260/280^1^**	**OD 260/230^1^**	***Ccoli*^2^ Ct values**	**DNA concentration^1^ (ng/μl)**	**Total DNA yield^1^ (μg)**	**PRNP^2^ Ct values**
**Nucleospin Blood (100)**	1.74 (0.02)^a, d^	1.79 (0.07)^a^	29.61 (0.17)^a^	56 (22.5)^a^^,^^d^	5.6 (15.9)^a^	24.95 (0.41)^a^
**Nucleospin Blood L (200)**	1.63 (0.02)^e^	1.31 (0.07)^d^	29.60 (0.17)^a^	118 (22.5)^a^^,^^d^^,^^c^	23.6 (15.9)^a^^,^^b^	24.82 (0.41)^a^
**Nucleospin Blood XL (2,000)**	1.68 (0.02)^a^^,^^d^^,^^e^	1.30 (0.07)^d^	29.66 (0.17)^a^	162 (22.5)^c^	324.3 (15.9)^c^	23.42 (0.41)^c^
**Nucleospin Blood- Buffy Coat (150)**	1.94 (0.02)^b^	2.10 (0.07)^a^^,^^b^	29.60 (0.17)^a^	63 (22.5)^a^^,^^d^	12.6 (15.9)^a^^,^^b^	23.95 (0.41)^a^^,^^c^
**Nucleospin Tissue- Buffy Coat (150)**	1.79 (0.02)^d^	2.10 (0.07)^a^^,^^b^	29.49 (0.17)^a^	59 (22.5)^a^^,^^d^	8.9 (15.9)^a^	24.77 (0.41)^a^^,^^b^
**Modified Blood (200)**	1.84 (0.02)^a^^,^^b^^,^^d^	2.18 (0.07)^b^	29.55 (0.17)^a^	93 (22.5)^a^^,^^c^^,^^d^	18. 8 (15.9)^a^^,^^b^	23.73 (0.42)^b^^,^^c^
**Modified Tissue (200)**	1.85 (0.02)^a^^,^^b^^,^^d^	2.14 (0.07)^a^^,^^b^	29.57 (0.17)^a^	120 (22.5)^a^^,^^c^^,^^d^	24.6 (16.4)^a^^,^^b^	23.23 (0.41)^c^
**Modified Dx (200)**	1.80 (0.03)^a^^,^^b^^,^^d^	2.08 (0.08)^a^^,^^b^	29.65 (0.18)^a^	289 (25.9)^b^	57.8 (18.4)^b^	22.95 (0.46)^c^
**Phenol-Chloroform (50)**	1.65 (0.03)^a^^,^^e^	1.98 (0.08)^a^^,^^b^	29.74 (0.18)^a^	258 (25.9)^b^	12.8 (18.4)^a^	24.69 (0.48)^a^
**Charge-Switch (250)**	1.49 (0.03)^c^	0.78 (0.08)^c^	29.64 (0.18)^a^	7.7 (25.9)^a^	1.8 (18.4)^a^	26.36 (0.46)^d^
**In-house (1,200)**	1.74 (0.03)^a^^,^^d^	1.87 (0.08)^a^^,^^b^	29.62 (0.18)^a^	302 (25.9)^b^	362.7 (18.4)^c^	23.32 (0.46)^c^

Two spectrophotometer measurements (OD260/280 and OD260/230 nm) and real-time PCR results of *C. coli* spiked samples (Ct values) used to assess DNA purity and PCR inhibitors respectively. DNA concentration was assessed with qubit measurements and real-time PCR targeting the PRNP gene (Ct values); results are marginal means with standard errors in parentheses.

^1^Higher values are desirable.

^2^Lower values are desirable.

^a,b,c,d,e^ Comparison of values within each column; values with the same superscript are not statistically different (P>0.05) from each other but they differ significantly (P<0.05) from values with different superscript.

### Spectrophotometric measurements

According to the 260/280 nm absorbance ratio results, the Nucleospin Blood-Buffy Coat protocol extracted the purest DNA, followed by Modified Tissue, Modified Blood and Modified Dx protocols (Table 3). These protocols showed a 260/280 nm ratio above 1.8, which is considered standard for pure DNA [26,30]. Moreover, Nucleospin Tissue-Buffy Coat, In-house and Nucleospin Blood protocols showed values near the desirable limit of 1.8. Differences among these seven protocols were not significantly different from zero (P>0.05) but they all led to significantly (P<0.05) purer DNA compared to Nucleospin Blood L and Charge Switch gDNA Tissue Mini Kit.

According to 260/230 nm ratio results (Table 3), Modified Blood, Modified Tissue, Modified Dx, Nucleospin Blood-Buffy Coat and Nucleospin Tissue-Buffy Coat protocols were associated with values above 2.0, which are the most desirable [30,38]. Phenol-Chloroform and In-house protocols followed with values slightly lower than 2. Although differences among these seven protocols were not significantly greater than zero (P>0.05), they all led to significantly (P<0.05) purer DNA compared to Nucleospin Blood L, Nucleospin Blood XL and Charge Switch Tissue Mini Kit protocols.

The two spectrophotometric measurements are frequently used for the evaluation of DNA purity [39–41]. A low 260/280 nm ratio is indicative of contamination with proteins, which could inhibit downstream applications and hamper DNA-banking [42]. A low 260/230 nm ratio is indicative of contamination with phenol or guanidine carried over during the washing steps of the silica columns or magnetic beads. Remaining organic compounds like phenol, guanidine, salt or solvents are also considered inhibitors for downstream applications [28].

According to the two spectrophotometric ratios, Nucleospin Blood L, Nucleospin Blood XL and Charge Switch Tissue Mini Kits did not extract pure DNA, suitable for NGS or microarray analysis. In the case of Nucleospin Blood L and XL kits this result could be attributed to the high volume of blood (2 and 10 ml, respectively) used for the extraction. All other methods that used high volumes of blood (commercial kits, Phenol-Chloroform, In-house protocol) had spectrophotometric measurements showing satisfactory purity levels. This improvement could be attributed to the addition of a buffy coat step which was an additional purification step from plasma proteins and erythrocytes. Therefore, isolated leucocytes seem to be more appropriate source of DNA compared to whole blood when high quantities of DNA recovery are required. However, in the case of Charge Switch Tissue Mini Kit, the buffy coat step was not adequate to extract pure DNA. The low purity of DNA in that case could be attributed to the fact that foam was formed during the washing steps. In the In-house developed protocol, foam formation during the washing steps was also a problem when ethanol was used in the washing buffers. The problem was solved by isopropanol instead of ethanol. Isopropanol was able to disperse and wash the magnetic beads more efficiently without the formation of foam.

### DNA concentration and total DNA yield

Concentration and total yield of extracted DNA were assessed using the Qubit Platform. Measurements revealed that the In-house protocol extracted the most concentrated DNA, followed by the Modified Dx and the Phenol-Chloroform protocols (Table 3). The differences among these three protocols regarding DNA concentration were not significantly greater than zero (P>0.05). Nevertheless, it should be mentioned that in the case of Phenol-Chloroform protocol, two extracts out of the sixteen had DNA concentration lower than 50 ng/μl, which is considered to be the minimum amount suitable for complex genotyping studies (http://genepool.bio.ed.ac.uk/illumina/samples.html). Moreover, these three protocols extracted significantly (P<0.05) more concentrated DNA compared to all other protocols used in the present study. Charge Switch Tissue Mini kit extracted DNA with an average concentration lower than 50 ng/μl while Nucleospin Blood, Nucleospin Blood-Buffy Coat and Nucleospin Tissue-Buffy Coat methods yielded average concentrations just over this threshold. However, many of the samples from these three methods had concentrations lower than 50 ng/μl. The introduction of modifications to Blood and Tissue kits increased considerably (almost doubled) the concentration of DNA extracted from buffy coat and always yielded DNA with concentration above the desirable threshold (Table 2). These modifications reduced the impurities and lowered the viscosity of the aqueous solution added to the silica columns. In the case of unmodified protocols, the gelatinous mucus created during the lysis step, which contained DNA among others, had to be removed since it could not get through silica membranes and thus, a DNA quantity was inevitably lost. The same problem was also present when magnetic beads were used. In order to overcome this problem, we used less blood (4.5 instead of 9 ml) in the preparation of buffy coat for the In-house protocol. In addition, lysis buffers in the In-house protocol contained detergents, which resulted in the dissolution of the gelatinous mucus created during the lysis step and thus high quantity of DNA was able to bind to the magnetic beads. In the case of Charge Switch kit, where no effort to optimize the protocol was performed, it seems that most of DNA present in the gelatinous mucus was not able to be absorbed on the magnetic beads.

Total DNA yield extracted with each protocol was calculated by multiplying DNA concentration measured with the Qubit platform with final elution volume (Table 2). According to these calculations, the In-house protocol extracted the highest DNA yield followed by Nucleospin Blood XL kit (Table 2). Interestingly, in the aforementioned protocols, a large extraction volume (1,200 μl and 2,000 μl, respectively) was used compared to the other protocols (50–250 μl). Differences between these two kits were not significant but both protocols performed significantly better compared to all the others (Table 3). Among the other protocols, Modified Dx kit recovered the highest DNA yield. Nucleospin Blood and Charge Switch Tissue Mini kits extracted the lowest DNA quantity. For Nucleospin Blood, this was due to the low initial blood volume (200 μl) used for the extraction, while for Charge Switch Tissue Mini kits, probably due to the inability of lysis buffer and proteinase K to dissolve the gelatinous mucus formed by leucocyte pellet.

The Qubit assay is the method of choice for accurate estimation of DNA quantity for NGS or microarray applications (http://genepool.bio.ed.ac.uk/illumina/samples.html). Qubit platform provides a rapid, sensitive and accurate method for dsDNA quantification with minimal interference from RNA, protein, single stranded DNA (primers) or other common contaminants that affect UV absorbance [43]. According to Qubit measurements, the In-house, Nucleospin Blood XL and Modified DX protocols extracted sufficient DNA quantity for use in large-scale genotyping applications, even for de Novo sequencing, and for the creation of DNA banks for future use at the same time (Table 3). Moreover, Modified Tissue, Modified Blood, Nucleospin Blood-Buffy Coat, Nucleospin Blood L and Phenol-Chloroform protocols recovered adequate DNA quantity for NGS applications (Table 3). Nucleospin Tissue-Buffy Coat and Nucleospin Blood protocols recovered enough DNA for smaller scale NGS applications like directed or targeted re-sequencing and for microarray analysis (Table 3).

### Real-time PCR

Two real-time PCR analyses, one targeting the ovine PRNP gene and the other the *C. coli* glyA gene, were conducted in order to evaluate the presence of amplifiable DNA and asses PCR inhibitors in the extracts spiked with *C. coli*, respectively. The PRNP gene is present in the sheep genome and is responsible for controlling resistance to Transmissible Spongiform Encephalopathy in sheep. *C. coli* glyA is a single copy gene which is a known target for the reliable identification and quantification of *C. coli* in complex samples.

Marginal means for Ct PRNP values obtained by each extraction protocol are shown at Table 3. Lower Ct values are desirable since they are associated with larger amounts of amplifiable DNA. The Modified Dx, Modified Tissue and the In-house protocols gave the lowest mean Ct values, followed by Nucleospin Blood XL and Modified Blood protocols. The differences among these five protocols were not statistically significant (P>0.05). However, they were statistically better (P<0.05) when compared to Nucleospin Blood, Nucleospin Blood L, Nucleospin Tissue-Buffy Coat and Phenol-Chloroform protocols. Moreover, all the protocols performed significantly (P<0.05) better compared to Charge Switch gDNA Tissue Mini kit. In addition, two samples extracted with the Phenol-Chloroform protocol had unexpectedly high Ct values as could be seen by the high standard deviation and the range of values measured for this protocol (Table 1). The OD measurements for these two samples were close to the average for this protocol. Furthermore, Qubit measurements showed the existence of high DNA quantity (267 ng/μl) in one of these two samples. No amplification was detected in the non-template controls.

**Figure 1:**
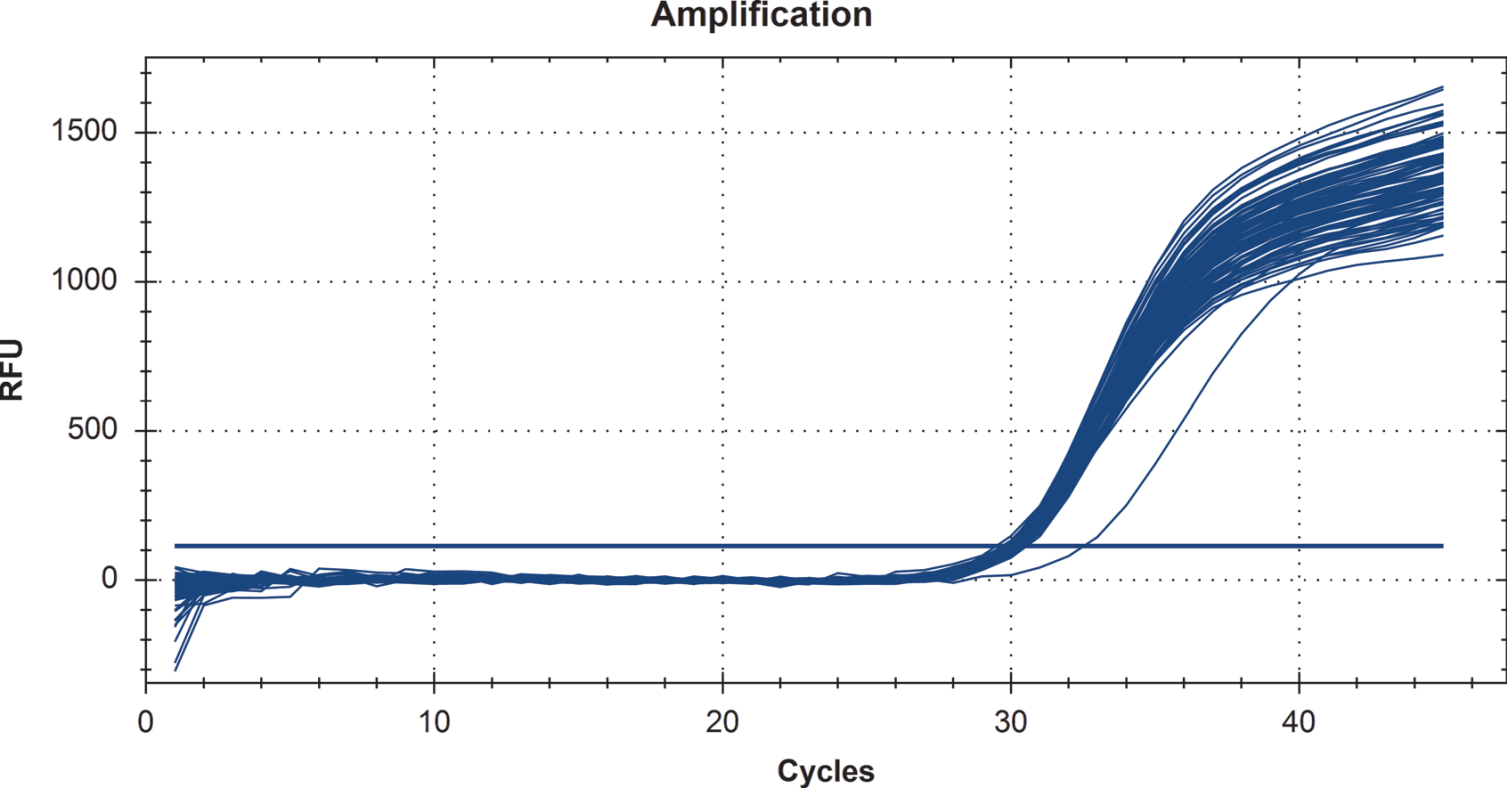
Real-time PCR amplification plot of the gly-A gene, from *Campylobacter coli* spiked extracted samples from sheep and the controls containing only the *Campylobacter coli* DNA spike. Results from one sample with higher Ct value, extracted with the Phenol-Chloroform protocol is also shown indicating the presence of inhibitors.

The real-time protocol targeting the gly-A gene of *Campylobacter coli*, showed an amplification efficiency of 99.9%. Marginal means for Ct values obtained after the addition of spiked genomic DNA are shown at Table 3. According to these results there was no statistically significant difference in the performance of the 11 DNA extraction protocols and the controls containing only the *C. coli* DNA spike, indicating efficient removal of PCR inhibitors. However, in the case of Phenol-Chloroform protocol, one sample showed an unexpectedly high Ct-value (Fig. 1), and another a very low fluorescence plateau; both samples had poor real-time PCR performance for the amplification of the PRNP gene. The first of these two samples was the one with the high DNA quantity, according to Qubit measurements. Furthermore, even though the OD ratios for this sample were not indicative of low purity DNA (OD 260/280 = 1.74 and OD 260/230 = 2.05), the real-time PCR results revealed the presence of PCR inhibitors.

In recent years, real-time PCR has become a reliable tool for assessing DNA quantity and quality for downstream applications since many of the large scale genotyping protocols include PCR based amplification steps [44–48]. The reason is that Ct values can assess PCR inhibition and utility of the extracted sample for molecular analysis. PCR analyses can be inhibited by compounds typically present in blood and co-extracted with the DNA. Therefore, we tested the DNA extracted with the 11 methods for the presence of PCR-inhibitors by using *C. coli* DNA spikes as an external control. None of the extracts caused a detectable inhibition, since no statistical significant differences were observed among the Ct values obtained when only spike DNA or spike and genomic sheep DNA (100 ng or 1000 ng), extracted with each one of the 11 methods, were present in the assay. The only exception was Phenol-Chloroform protocol (two samples), rendering it unsuitable for large-scale downstream applications.

### Gel Electrophoresis

**Figure 2:**
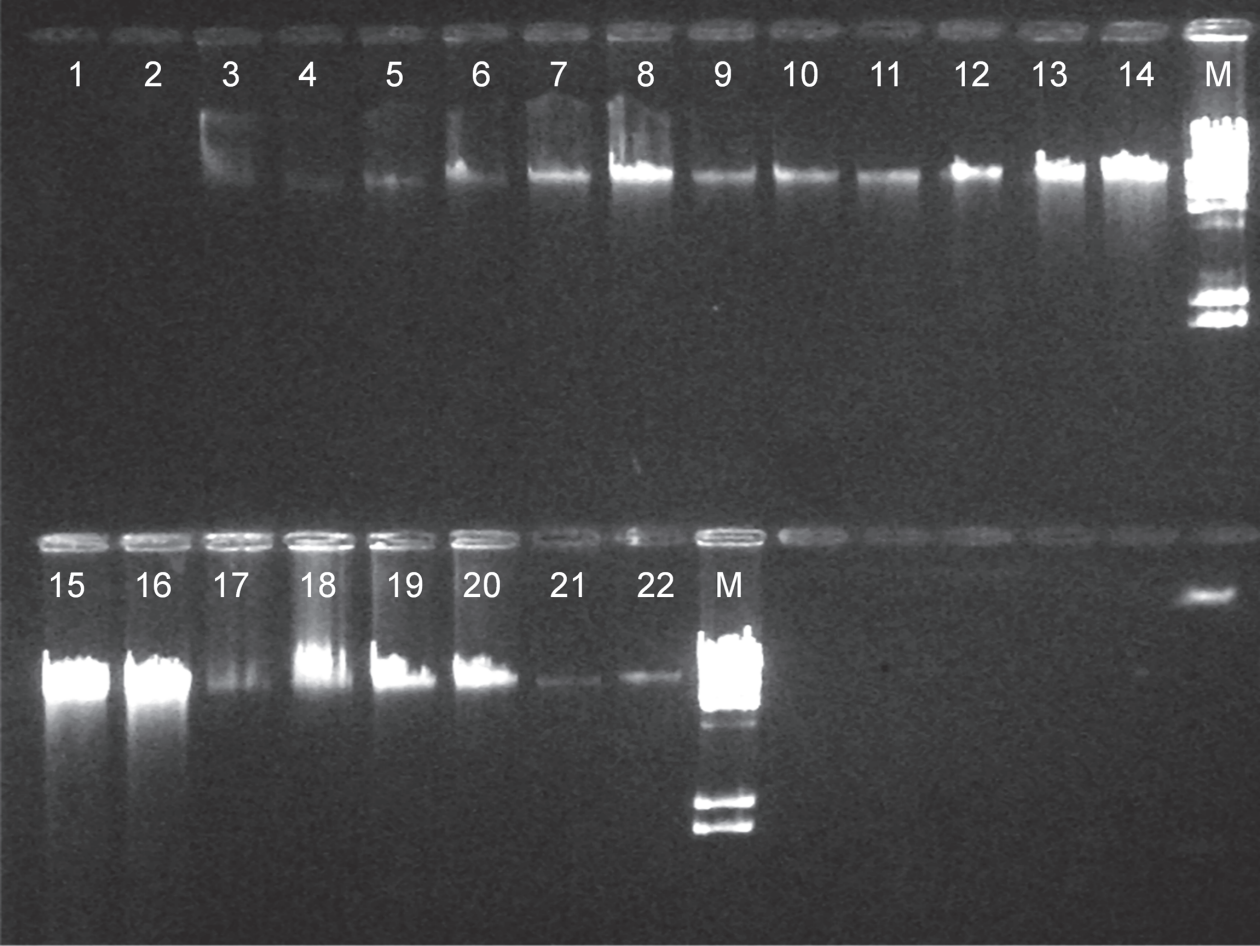
Representative results from gel electrophoresis analysis of genomic DNA from two different ovine blood samples extracted by eleven methods. Charge Switch gDNA Mini Tissue (lanes 1, 2), Nucleospin Blood (lanes 3, 4), Nucleospin Blood-Buffy Coat (lanes 5, 6), Modified Blood (lanes 7, 8), Nucleospin Tissue-Buffy Coat (lanes 9, 10), Modified Tissue (lanes 11, 12), Modified Dx (lanes 13, 14), Nucleospin Blood XL (lanes 15, 16), Phenol-Chloroform (lanes 17, 18), In-house (lanes 19, 20), Nucleospin Blood L (lanes 21, 22), M molecular weight marker l DNA/Hind III digest.

Integrity of the extracted DNA was assessed by agarose gel electrophoresis (Fig. 2). Gel electrophoresis revealed that high-molecular-weight non-degraded genomic DNA was obtained with all methods.

### Time, Labor and Cost Analysis

Comparison of the 11 protocols for labor intensity, throughput time and material cost per sample is reported in Table 4. The most rapid extraction method was the Nucleospin Blood kit while the most time-consuming was the Phenol-Chloroform protocol. The other commercial kits, modified or not, and the In-house developed protocol had intermediate time requirements. However, most of the required time involved no hands-on activities (e.g. longer incubation time with proteinase K). On the other hand, Phenol-Chloroform protocol was the cheapest, followed by the In-house developed and the Nucleospin Blood kit. The Charge Switch gDNA Tissue Mini kits, Nucleospin Blood L and Nucleospin Blood XL had an increased cost per sample by a factor of two, three and six, respectively, compared to the In-house protocol or the Nucleospin Blood kit. Moreover, the Modified protocols, Blood, Tissue and Dx were only slightly more expensive (0.2 euro per sample) compared to the unmodified ones. Nevertheless, DNA extraction by Modified Dx and Tissue kits cost 1 euro more compared to the In-house protocol. On the other hand, the In-house protocol required additional labor for the preparation of lysis and washing buffers. The Phenol-Chloroform protocol was the most technically difficult to perform and also required the use of highly toxic phenol. All other methods had almost the same labor intensity.

**Table 4:** Assessment of consumables cost per sample and process duration of 11 DNA extraction methods.

**Method**	**Process duration (24 samples)**	**Cost per sample (€)**
**Nucleospin Blood**	1.15 h	2.8
**Nucleospin Blood L**	2.5 h	9.3
**Nucleospin Blood XL**	2.5 h	18.45
**Nucleospin Blood-Buffy Coat**	3 h	2.8
**Nucleospin Tissue-Buffy Coat**	3 h	3.8
**Modified Blood**	3.15 h	3.0
**Modified Tissue**	3.15 h	4.0
**Modified DX**	3.15 h	3.9
**Phenol-Chloroform**	3 days	2.5
**Charge-Switch**	2.5 h	6.4
**In-house**	2.5 h	2.8

### Overall Evaluation of the 11 methods

Considering all criteria described in the present study, the most robust protocols in terms of DNA purity, concentration and total yield were the In-house and the modified versions of the commercial kits (Modified Blood, Modified Tissue and Modified Dx protocols). Though Nucleospin Blood, Nucleospin Blood-Buffy Coat and Nucleospin Tissue-Buffy Coat kits extracted very pure DNA, the quantity of DNA recovered showed high variation among samples, as could be seen by the high standard deviation and the other descriptive statistics presented in Table 2. In addition, the total DNA yield was not sufficient enough. On the other hand, Nucleospin Blood L and XL kits generated high yields of well concentrated intact DNA but with a barely passable purity level, as could be also depicted by the high standard deviation and the other descriptive statistics presented on Table 1. In addition, their high costs per sample rendered them unsuitable for large-scale high throughput applications. The Phenol-Chloroform protocol yielded highly concentrated DNA but in some cases DNA pellet was lost and PCR inhibitors were present (Tables 1 and 2, Fig. 1). Moreover, this method was laborious and prone to technical variation due to the multi-step protocol, limiting its suitability for large-scale studies. Finally, Charge Switch gDNA Tissue Mini kit had the poorest performance by all criteria considered.

### Large-Scale Application, Long-Term DNA Storage and Microarray Genotyping

**Figure 3:**
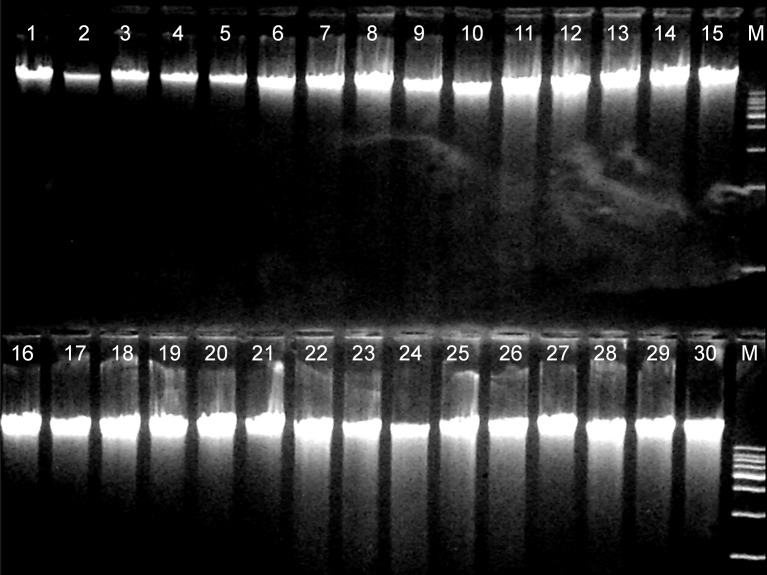
Representative results from gel electrophoresis analysis of genomic DNA from thirty different ovine blood samples extracted by four methods. Modified Blood (lanes 1 to 8), Modified Tissue (lanes 9 to 15), Modified Dx (lanes16 to 22), In-house (lanes 23 to 30), M molecular weight marker l DNA/Hind III digest.

Based on the above overall evaluation, the Modified Blood, Tissue and Dx kits, and the In-house protocol were selected to be used for DNA extraction of 600 ovine blood samples from as many individual animals. These DNA extracts were tested for their suitability for long-term storage and large-scale application. Gel electrophoresis of DNA extracts revealed specific clear bands with adequate quantity, suitable for microarray analysis, after three years of storage in −20°C (Fig. 3). No deterioration of DNA during the long storage was observed in any of these extracts, according to gel electrophoresis and Qubit measurements. The purity scores of genomic DNA extracted by the Modified Blood, Tissue and Dx kits, and In-house protocols, after three years of storage in −20°C, are summarized in Table 5. According to the Nanodrop measurements all protocols extracted pure DNA, with OD 260/280 above 1.8 and OD 260/230 above 2.0. The variability among samples within the same protocol was very low as shown by the low standard error estimates. Despite their statistical significance (P<0.05), differences observed were not practically relevant since all four protocols clearly exceeded the threshold values for DNA purity. Moreover, all four protocols extracted sufficient quantity of highly concentrated (above the 50 ng/μl threshold) DNA suitable for complex whole-genome genotyping and DNA-banking from all 600 animals (Table 5). Specifically, Modified Dx protocol extracted the most concentrated DNA, followed by the other two modified protocols (Modified Blood and Tissue, Table 5). The three modified protocols had performed, on average, better regarding the quantity of the DNA extracted compared to the results obtained from the initial evaluation, based on the samples from the 16 ewes (Table 3). On the other hand the In-house protocol extracted less concentrated DNA compared to the modified protocols but it still recovered multiple times more DNA, due to the high elution volume used (6 times higher compared to the modified kits).

**Table 5:** Statistical comparison of four genomic DNA extraction methods based on spectrophotometer measurements (OD260/280nm, OD260/230nm), DNA concentration and Total DNA yield; results are marginal means with standard errors in parentheses pertaining to 600 animals.

**Method**	**OD260/280**	**OD 260/230**	**DNA concentration(ng/ μl)**	**Total DNA yield (μg)**
**Modified Blood**	1.85 (0.01)^a^	2.19 (0.03)^a^	218 (14)^a^	43 (4.9)^a^
**Modified Tissue**	1.90 (0.01)^b^	2.40 (0.03)^b^	248 (14)^a^	49 (5.0) ^a^
**Modified Dx**	1.89 (0.01)^b^	2.35 (0.02)^b^	310 (08)^b^	62 (2.9)^a^
**In-house**	1.85 (0.01)^a^	2.17 (0.04)^a^	207 (17)^a^	237 (6.2)^b^

^a,b^ Comparison of values within each column; values with the same superscript are not statistically different (P>0.05) from each other but they differ significantly (P<0.05) from values with different superscript.

All extracts from the 4 methods proved to be equally suitable for microarray genotyping applications, based on the SNP call rates obtained. Table 6 summarizes results from the assessment of genotyping quality by DNA extraction protocol. The marginal means of average and individual SNP call rates per protocol were the same, showing that the variation of individual SNP call rate within the same animal does not affect results. No statistical significant differences of the effect of the four extraction protocols on microarray genotyping were detected (Table 6). These results were expected, since in all cases an adequate quantity of very pure and well concentrated DNA was provided for the genotyping.

**Table 6:** Statistical comparison of four genomic DNA extraction methods applied to 600 animals, based on microarray genotyping quality using average call rates across all SNP and individual SNP call rates; marginal means with standard errors in parentheses.

**Protocol**	**Average Call Rates**	**Individual SNP Call Rates**
**Modified Blood**	0.775 (0.003)^a^	0.775 (0.0012)^a^
**Modified Tissue**	0.775 (0.003)^a^	0.775 (0.0013)^a^
**Modified Dx**	0.775 (0.003)^a^	0.775 (0.0008)^a^
**In-house**	0.777 (0.004)^a^	0.777 (0.0016)^a^

^a^ Comparison of values within each column; values with the same superscript are not statistically different (P>0.05) from each other.

## Conclusions

Eleven extraction protocols were examined for effectiveness and efficiency in extracting and purifying high quantity of genomic DNA from blood samples, suitable for large–scale complex genotyping analysis and long-term DNA-banking. Four protocols, namely the In-house developed, and Modified Dx, Modified Tissue and Modified Blood appeared to be the best choices to extract high yields of pure highly concentrated genomic DNA from buffy coat. Ovine blood was used here as a study model but results may be generalized to humans or other mammalian species.

In general, DNA requirements for microarray analysis can be met with the use of most of the commercially available extraction kits. The most common problem when blood or tissue extraction kits, like Nucleospin Blood, are applied is that in many cases less than required concentrated DNA is extracted (<50 ng/μl). Therefore, the whole extraction procedure should be repeated. Moreover, according to the results of this study, simple commercial extraction kits are not sufficient to extract the quantity of DNA needed for NGS applications or for the creation of DNA banks. For these cases, there are other available commercial kits, like Nucleospin Blood L and XL. However, these kits which are quite expensive and, as shown in this study, there is a shortcoming regarding the purity of the extract. The four proposed protocols, developed, evaluated and tested for microarrays genotyping can provide very high quantities of pure DNA at a relatively low cost without being excessively laborious, rendering them appropriate for whole-genome large-scale applications. The fact that these four protocols were based on buffy coat instead of whole blood exemplifies the importance of the former in DNA extraction.

As NGS applications become routine in the near future, all the four proposed protocols might provide very handy solutions. In addition, extracting quite higher DNA quantities than needed for present applications and creating DNA banks can be very useful for follow-up or parallel research projects. Visiting farms, collecting individual blood samples and extracting DNA can be an expensive, demanding and laborious procedure which, if minimized, could yield great savings in time and resources. In addition, extraction of high quality DNA of precious, difficult to collect samples, which can be archived for future reference, would be extremely useful.
